# Remarks on Dr. Parry's Paper

**Published:** 1817-02

**Authors:** George Kerr


*For the London Medical and Physical Journal.*

### *Remarks on Dr. Parry's Paper;* by GEORGE KERR, Esq.

I AM much obliged to Dr. Parry for his correction of my statement concerning the descending aorta of the dog, tied by Mr. Astley Cooper, as described in the Medico-Chirurgical Transactions, Vol. II. I have again carefully examined the engraving and references, and, although the shoots from above are neither said nor indeed appear to enter directly into the part of the vessel below the ligature, it is, in my opinion, impossible that the bifurcation marked *e* and *g*, could have been in existence when the ligature was applied. If we suppose arteries given off above the ligature anastomozing with those below, how do we dispose of the conflicting currents?—How can we account for the exact co-apta-

 tion tion of the extremities of the vessels? I may be told, that there are no conflicting currents—that the lower part of the vessel is deprived of pulsation by the ligature; but, Harvey asserts, that collateral branches immediately, or very speedily, supply the functions of the vessel interrupted; and, if pulsation be restored in the main trunk, part of it remaining obliterated, the difficulty in accounting for the circulation by anastomozing branches remains, as far as I can see, in full force.

I have now seen Dr. Parry's very interesting work on the arterial pulse, in which he gives a beautiful delineation of the appearances on dissection of the left carotid of a ram, tied on the 27th of September, 1815, (I suppose 1814,) and the animal killed on the 7th of August following. The trunk of the vessel appears quite obliterated, and fine new arterial shoots appear connecting the parts of the vessel on each side of the obliteration. In reference to the plate ii. fig. 2, Dr. Parry says, pp. 160, 161—"This is merely an enlarged and somewhat different outline view of the *newly-generated* vessels, &c." "To this conclusion, as to the *new creation of vessels* in the case before us, objections have been raised with an ardour which has certainly reached the utmost limits of philosophical candour. Thus it has been argued: first, that these communicating branches must have previously existed, although they were not noticed. This is an objection that could be only made by those who are unacquainted with the structure and relations of the carotid arteries in sheep." He explains how easily these may be separated from their connections, and adds, "under these circumstances, it would be absurd to suppose, that a plexus of vessels could ever be mistaken for a single trunk; or that these vessels, projecting as they do all around from the main line of the artery, could have been separated from the surrounding parts without being wounded by the knife. But it is said, they were too small to discharge blood, and only became enlarged, when their enlargement was rendered necessary by a ligature." In what mode, however, a ligature could be applied to a round large vessel included within a plexus of collateral arteries, without that plexus being either cut or seen, it is for those to explain or conceive who make the objection."

To me it appears, that Dr. Parry proves to full demonstration the production of new vessels passing over the part of the main trunk obliterated and again connecting themselves with it; and I still think, that, when Mr. Astley Cooper tied the aorta of the dog already mentioned, no such vessels could have been in existence as the engraving represents.

sents.* I am glad to find, that what appears a great difficulty to me, viz. how the animal can exist for the first days, or even hours, after the operation, appears also a difficulty to Dr. Parry, who is so well qualified, and possesses such opportunities, of ascertaining the truth by experiments. I confess that the fact appears absolutely irreconcileable with our received notions of the circulation of the blood, for the reasons assigned in my first letter; and, I look upon Dr. Parry's work alluded to as most important, because it must lead to further investigation.

I do not collect, from Dr. Parry's experiments, that he attended particularly to the size of the vertebral arteries in cases where the carotids have been obliterated; and, I think it is of some importance to suggest this, because large arteries have been tied in situations where it appears to me altogether impossible that collateral vessels can perform, even after a lapse of time, the office of the vessel obliterated.

The notion of Scarpa and others, that arterial branches performing a supposed vicarious duty, become tortuous from rapidity of motion, seems altogether unphilosophical. Rapidity of motion ever tends to the rectilinear, and, I observe, Dr. Parry illustrates this by reference to an experiment, p. 131. A warm injection thrown into the dead subject will necessarily occasion twisting, or contortion; but that, I presume, proceeds from the partial application of heat, as many substances turn and twist when thrown upon the fire. Let us hope that Dr. Parry will prosecute his experiments, for, I am well assured, that he will follow the truth wherever it may lead him, even if it should induce him to question the infallibility of Harvey.

*Aberdeen; Nov.* 17, 1816.

---

*For the London Medical and Physical Journal.*

### *On Tinea Capitis;* by L. G.

IT is said by a celebrated teacher in one of our schools of surgery, that there is no sure remedy for tinea capitis. —This saying is probably true; at the same time, a certain degree

---

* The vessels given off at the part of the artery tied by Mr. Cooper, go off at nearly right angles, as small branches proceeding from large vessels generally do. The view given represents two large branches passing off at very acute angles, and coming in close contact with the aorta immediately below the ligature.

